# Obesity Is Independently Associated with Spinal Anesthesia Outcomes: A Prospective Observational Study

**DOI:** 10.1371/journal.pone.0124264

**Published:** 2015-04-21

**Authors:** Hyo-Jin Kim, Won Ho Kim, Hyung Woo Lim, Jie Ae Kim, Duk-Kyung Kim, Byung Seop Shin, Woo Seog Sim, Tae Soo Hahm, Chung Su Kim, Sangmin M. Lee

**Affiliations:** 1 Department of Anesthesiology and Pain Medicine, Samsung Changwon Hospital, Sungkyunkwan University School of Medicine, Changwon, Republic of Korea; 2 Department of Anesthesiology and Pain Medicine, Samsung Medical Center, Sungkyunkwan University School of Medicine, Seoul, Republic of Korea; Montclair State University, UNITED STATES

## Abstract

The influence of body-mass index (BMI) on spinal anesthesia is still controversial, with discrepant results reported in previous studies. To compare spinal anesthesia in obese and non-obese subjects, the anesthesia profiles in patients who underwent spinal anesthesia using intrathecal hyperbaric bupivacaine were compared. A total of 209 patients undergoing elective total knee replacement arthroplasty (TKRA) surgery under spinal anesthesia were divided into an NO (non-obese) group (BMI < 30 kg/m^2^, n = 141) and an O (obese) group (BMI ≥ 30 kg/m^2^, n = 68). Anesthesia was deemed successful if a bilateral T12 sensory block occurred within 15 minutes of intrathecal drug administration, and if the level of sensory block was higher than T12 when the surgery ended. Logistic regression analysis with multiple variables known to influence spinal anesthesia was performed to identify which parameters independently determined the spinal anesthesia outcome. Similar doses of bupivacaine were administered to the NO and O groups. The incidence of anesthesia failure was significantly lower in the O group [n = 43 (30.5%) in the NO group vs. n = 10 (18.9%) in the O group, p = 0.014]. The independent predictors for successful anesthesia in all patients were dose of hyperbaric bupivacaine [odds ratio (OR) 2.12, 95% CI: 1.64–2.73] and obese status (BMI ≥ 30 kg/m^2^, OR 2.86, 95% CI: 1.25–6.52). Time to first report of postoperative pain and time to first self-void were significantly longer in the O group. These results suggest that the duration of block with hyperbaric bupivacaine is prolonged in obese patients and obesity is independently associated with spinal anesthesia outcomes, as is bupivacaine dosage. A further study enrolling patients with morbid obesity and using a fixed bupivacaine dosage is required to confirm the effect of obesity on spinal anesthesia.

## Introduction

The spread of spinal anesthetic drugs has been reported to be variable [1]. The sensory block level in patients undergoing spinal anesthesia can be influenced by numerous patient demographic factors including age, gender, height, weight, body-mass index (BMI), spinal anatomy, and lumbosacral cerebrospinal fluid (CSF) volume [2–9]. However, the influence of BMI on spinal anesthesia is still controversial, with discrepant results reported in the literature. Since the prevalence of obesity in the operation room is increasing, it is important to investigate the impact of obesity on spinal anesthesia profiles [10,11].

Many previous studies have investigated the effect of obesity on spinal anesthesia, but the results have not been consistent [3–6,9,12–15]. The results differ according to the baricity of the local anesthetics used for spinal anesthesia and the study outcome variable chosen for comparison between obese and non-obese patients. Previous studies that compared maximal cephalad spread with isobaric local anesthetics have reported positive relationships between obesity and sensory block level [3–6,15]. This increased spread has been assumed to be due to the reduced CSF volume caused by large amounts of epidural fat or extradural vein distention [9,12]. However, blockade height can also be influenced by the baricity of local anesthetics. A previous study demonstrated that isobaric local anesthetics showed variable block heights [1]. With hyperbaric bupivacaine, no significant difference in block level between obese and non-obese patients was observed in a previous study [3]. Meanwhile, recent dose-response studies compared median effective dose (ED_50_) for successful anesthesia between obese and non-obese patients, reporting no difference [13,14]. However, these previous results were limited mainly to maximal block height, not block duration. Reduced CSF volume might also influence the block duration, but there has been no study comparing the block duration between obese and non-obese patients.

Thus, the influence of obesity on spinal anesthesia is still unclear. Moreover, no definite guidance is available regarding whether doses of spinal anesthetics can be reduced in obese patients. On the one hand, spinal anesthesia that is shorter than the duration of surgery yields anesthesia failure. On the other hand, spinal anesthesia prolonged beyond surgery time can cause patient discomfort, bladder dysfunction, and/or long hospital stays. Since obese patients often undergo outpatient surgery for varicose veins or inguinal hernia, it is important to investigate the spinal anesthesia characteristics of obese patients. Delayed recovery of spinal anesthesia can result in longer stays in the outpatient surgery unit and unplanned hospitalizations, which can result in patient dissatisfaction and increased medical costs. Therefore, it is important to determine whether obesity influences spinal anesthesia outcomes, particularly anesthesia duration.

The aim of this prospective observational study was to determine whether or not the duration of spinal anesthesia differs among obese and non-obese patients. To achieve this goal, we compared neuraxial blockade levels of the two groups at the anesthesia induction and at the end of surgery in similar settings. Although many factors influence spinal anesthesia outcomes, previous studies seldom applied multivariate analysis to find independent determinants for spinal anesthesia outcomes. Therefore, logistic regression analysis with multiple variables known to influence spinal anesthesia spread such as age, gender, dosage of local anesthetics and spinal column length, in addition to obesity, was performed to identify which parameters determined the final anesthesia level at the end of surgery.

## Materials and Methods

### Design and Subjects

This prospective observational study was approved by the Samsung Medical Center Institutional Review Board (SMC-2012-04-089), and all patients provided written informed consent. Patients who were classified as ASA physical status I-III (S1 Table) and who were scheduled for total knee replacement arthroplasty (TKRA) at Samsung Medical Center between May 2012 and December 2012 were consecutively enrolled in this study. This study was registered at http://www.clinicaltrials.gov (NCT01609517). Patients with diabetes mellitus or other neuropathies, a history of previous spinal surgery, a skin infection at the site of injection, an allergy to bupivacaine, or another contraindication to spinal anesthesia such as hypovolemia, circulatory shock, increased intracranial pressure, coagulopathy, or sepsis were excluded. According to the obesity criteria issued by a panel of experts from the World Health Organization [16], patients with a BMI < 30.0 kg/m^2^ were assigned to the non-obese group (NO group, n = 141), and those with a BMI ≥ 30.0 kg/m^2^ were assigned to the obese group (O group, n = 68). Patient characteristics, including age, gender, and clinical parameters, are presented in Table 1. Anesthesiologists and outcome assessors were aware of the group assignment, but data analysts were blind to the BMI group of each patient.

**Table 1:** Patient characteristics and clinical parameters.

	Group NO	Group O	p-value
**Age (years)**	72 (65–75)	72 (68–77)	0.418
**Height (cm)**	150 (147–154)	150 (147–153)	0.246
**Weight (kg)**	56 (53–62)	76 (72–82)	<0.001
**BMI (kg/m** ^**2**^ **)**	25.7 (23.3–28.1)	33.8 (32.4–35.7)	<0.001
**Gender (male/female; n)**	19 / 122	9 / 59	0.962
**Vertebral column length, cm**	58 (50–64)	56 (49–61)	0.069
**ASA (I/II/III; n)**	29 / 87 / 25	13 / 46 / 9	0.646
**Bupivacaine dosage, mg**	8 (7–10)	8 (7–10)	0.863
**Duration of surgery (min)**	109 (101–115)	106 (95–115)	0.116
**Tourniquet time (min)**	95 (85–107)	90 (82–102)	0.108

The values are presented as the median (interquartile range), or the number of patients (%) per group.

Group NO = non-obese group; Group O = obese group; BMI = body-mass index; ASA = American Society of Anesthesiologists Physical Status.

### Study Protocol

Patients did not receive any premedication. The BMI of each patient was calculated using the weight and height values obtained in our hospital [17]. Ward nurses were responsible for anthropomorphic measurements of the patients. The weight (kilograms) and height (meters) were measured with the patient in a standing position using an automatic anthropometer the evening before the day of surgery. Patients fasted before dinner and wore a hospital gown. The BMI values were calculated by SPSS software.

Standard monitoring was applied in the operating room. All patients were administered 8 ml/kg of lactated Ringer’s solution during the first 10 minutes of spinal anesthesia. A maintenance dose of 4 ml/kg/h was continually administered subsequently thereafter. Further, 8 ml/kg of colloid (Voluven, Fresenius Kabi, Germany) was administered at 6 ml/kg/h during surgery. Oxygen was given at 5 L/min via a face mask during surgery. Spinal anesthesia was performed with the patient in the lateral position. Spinal puncture was performed at the L4-5 interspace using a 25 gauge Whitacre spinal needle (Kimberly-Clarke, Roswell, GA, USA) [18]. A freshly prepared solution of hyperbaric bupivacaine (Marcaine Spinal, AstraZeneca AB, Sodertalje, Sweden, 5 mg/ml) was injected over 20 seconds. The dose of bupivacaine was left to the discretion of the attending anesthesiologists. In all cases, a 2 ml syringe was used. Moreover, no epinephrine or fentanyl was added to the bupivacaine solution. The needle orifice faced cephalad during injection, and all patients were then placed in a supine horizontal position.

Successful anesthesia induction was defined as a bilateral T12 sensory block to pinprick within 15 minutes of intrathecal drug administration and a sensory/motor block scale ≥ 2. Successful anesthesia for TKRA was defined as successful anesthesia induction coupled with a ≥ T12 level of sensory block at the end of the surgery, with a sensory/motor block scale ≥ 2 [19]. Anesthesia failure was defined as either an induction failure or a sensory block lower than T12 at the end of the surgery. These definitions were modified from previous definitions of similar studies, focusing on the duration of spinal anesthesia and the blockade of tourniquet pain [13,14,20]. Successful anesthesia was set as the endpoint in the multivariate logistic regression analysis. Tourniquet pain was also assessed at the end of surgery. Tourniquet pain was recorded if the reported pain score was ≥ 2 (numerical rating scale in which 0 represented “no pain” and 10 represented “worst pain imaginable”) [21]. If a patient reported surgical or tourniquet pain during surgery, remifentanil (0.05–0.3 μg/kg/min) was infused for pain control. Subjects with a failed induction (n = 9) were excluded from the analyses of variables related to spinal anesthesia characteristics and the incidence of adverse events. There was no missing data except subject with a failed induction.

### Measurements

Vertebral column length was measured from the spinous process of the seventh cervical vertebra to the sacral hiatus with the patient in the lateral position before spinal anesthesia. Sensory and motor block levels were determined at 5 minutes intervals until 20 minutes after intrathecal drug administration, and were determined again at the end of the surgery. Sensory levels to pinprick were assessed using the Hollmen scale [19]: 0 = an ability to appreciate a pinprick as sharp, 1 = the perception of a pinprick in blocked areas as less sharp than in unblocked areas, 2 = the perception of a pinprick as a touch but not as sharp (analgesia), and 3 = an inability to feel a pinprick (anesthesia). Sensory blocks were recorded bilaterally along the mid-clavicular line by assessing pinprick sensations using a 25 G needle. The lower block level was selected if the sensory block level was different on both sides. Motor block in the lower limbs was graded using the Bromage scale [22]: 0 = the ability to lift an extended knee at the hip, 1 = the ability to flex the knee but not to lift an extended leg, 2 = the ability to only flex the toes, and 3 = the inability to move the hips, knees, and toes.

Hypotension was defined as a decrease in systolic arterial pressure ≥ 20% relative to the average preoperative value, or a mean arterial pressure < 55 mmHg. When hypotension occurred, repeated intravenous ephedrine bolus doses of 5 mg were administered. If the heart rate was < 50 beats/min or 20% lower than the measured preoperative value, atropine (0.5 mg) was administered. Time to first report of postoperative pain and time to first self-void were measured from the end of surgery in all cases.

### Statistical Analysis

Sample size was determined under the assumption that the expected odds ratio of obesity for successful anesthesia would be 4.0 [23]. We estimated that 175 patients or more would be required to detect a 30% difference in success rate with a power of 0.8 and a type I error rate of 0.05. To compensate for drop out, we intended to recruit 200 or more patients.

Sample size was also validated by the suggestion that the result of a multivariate logistic regression model should have more than 10 outcome events per independent predictor for accuracy [24]. Thus, an entire population of 200 patients or more was estimated to be required to permit unbiased fitting of five or fewer independent predictors in a multivariate logistic regression model (with an estimated 25% incidence of anesthesia failure, as defined in this study) [24].

All statistical analysis was performed with SPSS software (version 20.0, SPSS Inc., Chicago, IL, USA). Categorical variables are reported as absolute numbers (*n*) and relative frequencies (%), whereas continuous variables are reported as means (standard deviation) or medians (interquartile ranges), as appropriate. Continuous data were compared using the Mann-Whitney U-test. Fisher’s exact test or the Chi-square test was used to compare categorical variables according to expected count. Logistic regression models were used to identify univariate and multivariate predictors of successful spinal anesthesia in the entire population. Univariate logistic regression analysis was first used to identify possible predictors of successful anesthesia. The multivariate model included only variables that were found to be significant by univariate analysis (p<0.05). Predictor variables were selected from a list of candidate variables by performing a backward Wald selection with a significance criterion of p<0.05. Body-mass index was included in the analysis both as a categorical and continuous variable. Cox proportional hazards regression models and log rank tests were used to compare time to first report of postoperative pain and time to first self-void. Presented p-values are nominal p-values, unadjusted for the number of tests being performed.

## Results

**Fig 1:**
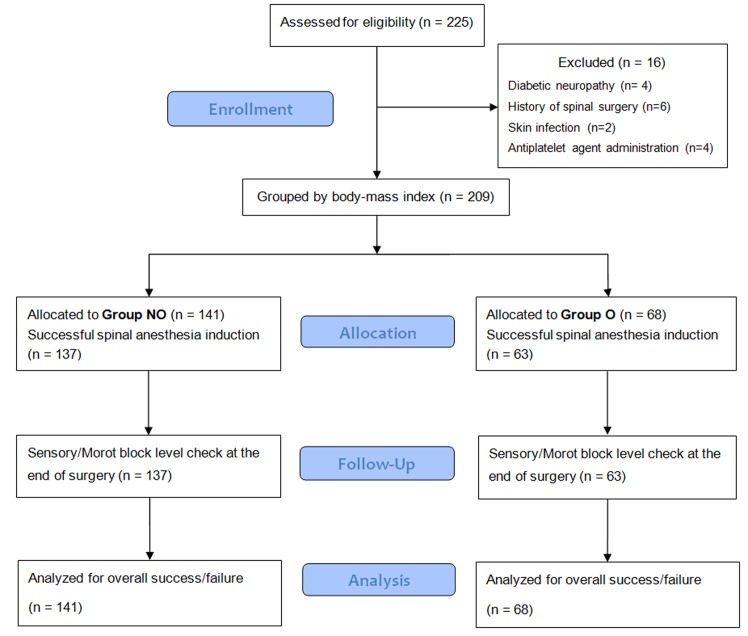
Flow diagram outlining the study procedure.

**Fig 2:**
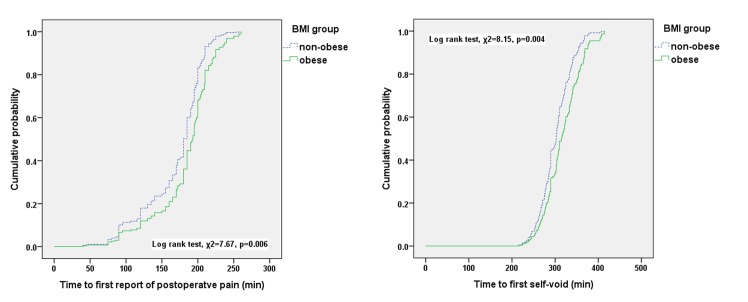
Comparison of time to first report of postoperative pain and time to first self-void. P-values are obtained using log-rank tests.

During the study period, 225 patients were assessed for eligibility. Of these, 16 patients were excluded from the study due to diabetic neuropathy (n = 4), a history of previous spinal surgery (n = 6), skin infection at the site of injection (n = 2), or antiplatelet agent administration (n = 4). The remaining enrolled 209 patients completed the study according to the protocol and were included in the analysis (Fig 1). With the exception of body weight and BMI, all demographic and clinical parameters were similar between groups (Table 1). Similarly, the bupivacaine dosages administered were not different between the two groups. The characteristics of spinal anesthesia and incidences of adverse events in the two groups are compared in Table 2. The incidence of anesthesia failure (according to the definition used in this study) was significantly lower in the O group [n = 43 (30.5%) in the NO group vs. n = 10 (14.7%) in the O group, p = 0.014]. The incidence of anesthesia induction failure was not different between the two groups. Sensory block levels at anesthesia induction and at the end of surgery were significantly higher in the O group compared with the NO group [induction: T9 (T11-T7) in the NO group vs. T8 (T10-T6) in the O group, p = 0.031]. No patients in either group reported surgical pain during the surgery. Incidences of tourniquet pain, as well as incidences of adverse events related to spinal anesthesia, were not different between the two groups. Time to first self-void and time to first report of surgical pain were significantly longer in the O group compared with the NO group according to the Log-rank test (p = 0.006, 0.004, respectively, Fig 2).

**Table 2:** Characteristics of spinal anesthesia and incidence of adverse events.

	Group NO	Group O	p-value
**Success/failure of anesthesia (n/n)** ^**a**^	98 / 43 (30.5%)	58 / 10 (14.7%)	0.014
**Success/failure of anesthesia induction (n/n)** ^**a**^	137 / 4 (2.8%)	63 / 5 (7.4%)	0.154
**Peak level of sensory block at anesthesia induction**	T9 (T11—T7)	T8 (T10—T6)	0.031
**Time to peak sensory block (min)**	10 (10–15)	10 (10–15)	0.160
**Maximum Bromage Scale 0-1-2-3 (n)**	0–0–103–34	0–0–41–22	0.175
**Incidence of tourniquet pain (n, %)**	17 (12.4%)	5 (7.9%)	0.467
**Level of sensory block at the end of surgery**	T12 (L2—T11)	T11 (T12—T11)	0.046
**Incidence of hypotension (n, %)**	7 (5.1%)	5 (7.9%)	0.523
**Total dose of ephedrine (mg)**	5 (5–6.25)	5 (5–7.5)	0.876
**Baseline MBP (mmHg)**	62 (58–67)	61 (56–67)	0.467
**Lowest MBP (mmHg)**	56 (54–60)	56 (54–59)	0.365
**Decrease in MBP (mmHg)**	6 (4–7)	6 (2–7)	0.985
**Incidence of bradycardia (n,%)**	9 (6.6%)	9 (14.3%)	0.108
**Incidence of nausea (n, %)**	10 (7.3%)	5 (7.9%)	0.999
**Incidence of vomiting (n, %)**	3 (2.2%)	2 (3.2%)	0.651
**Incidence of shivering (n, %)**	10 (7.1%)	1 (1.6%)	0.178
**Time to first report of postoperative pain (min)**	180 (140–200)	190 (170–200)	0.006^b^
**Time to first self-void (min)**	300 (277–327)	315 (299–345)	0.004^b^
**Length of hospital stay (day)**	6 (5–7)	6 (5–8)	0.213

The values are presented as the median (interquartile range), or the number of patients (%) per group.

Group NO = non-obese group; Group O = obese group; MBP = mean blood pressure; decrease in MBP = baseline MBP—lowest MBP.

^a^ Other variables except these two were analyzed by using data of those with successful anesthesia induction.

^b^ p-values are the results of log rank test.

Univariate analysis of predictors of successful anesthesia in all patients revealed that weight, BMI ≥ 30 kg/m^2^, and bupivacaine dosage (mg) were significant predictors for successful anesthesia (Table 3, S2 and S3 Tables). Multivariate analysis of predictors of successful anesthesia in all patients showed that BMI ≥ 30 kg/m^2^ (odds ratio: 2.86 95% CI: 1.25–6.52) and bupivacaine dosage (odds ratio: 2.12, 95% CI: 1.64–2.73) were independently associated with anesthesia success (Table 4). When BMI was included in the analysis as a continuous variable, it was also independently associated with anesthesia success (odds ratio: 2.86, 95% CI: 1.04–1.22).

**Table 3:** Univariate analysis of predictors for successful anesthesia in all patients in the Group O and NO.

	Anesthesia success (n = 156)	Anesthesia failure (n = 53)	Odds Ratio (95% CI)	p-value
**Age (years)**	71 (65–76)	73 (67–78)	0.97 (0.93–1.01)	0.133
**Height (cm)**	150 (147–154)	149 (146–154)	1.02 (0.97–1.09)	0.350
**Weight (kg)**	65 (56–76)	57 (53–68)	1.04 (1.01–1.07)	0.008
**BMI ≥ 30 kg/m** ^**2**^	58 (37.2%)	10 (18.9%)	2.55 (1.19–5.45)	0.016
**BMI (kg/m** ^**2**^ **)**	28.5 (24.7–32.5)	26.3 (24.4–29.2)	1.10 (1.02–1.18)	0.012
**Female gender (n)**	132 (84.6%)	49 (92.5%)	0.45 (0.15–1.36)	0.157
**Spinal column length (cm)**	57 (51–63)	54 (48–62)	1.03 (0.99–1.07)	0.085
**Bupivacaine dosage (mg)**	9 (7–10)	7 (6–8)	2.08 (1.61–2.69)	<0.001

The values are presented as the mean (SD), median (interquartile range), or the number of patients (%) per group.

Group NO = non-obese group; Group O = obese group; BMI = body-mass index.

**Table 4:** Multivariate analysis of predictors for successful anesthesia in all patients in the group O and NO.

Covariate	β-coefficient	Odds Ratio	95% CI	p-value
***BMI as a categorical variable***				
**BMI ≥ 30 kg/m** ^**2**^	1.05	2.86	1.25–6.52	0.013
**Bupivacaine dosage (mg)**	0.75	2.12	1.64–2.73	<0.001
**Constant**	-5.12	0.006		<0.001
***BMI as a continuous variable***				
**BMI (kg/m** ^**2**^ **)**	0.12	1.13	1.04–1.22	<0.001
**Bupivacaine dosage (mg)**	0.76	2.15	1.66–2.78	<0.001
**Constant**	-8.20	0.000		<0.001

BMI = body-mass index, CI = confidence interval.

## Discussion

In this prospective observational study, a comprehensive comparison of spinal anesthesia-related parameters between non-obese and obese patients was performed to determine the influence of BMI on spinal anesthesia outcome. We recruited patients who underwent TKRA under spinal anesthesia and grouped these patients according to BMI. The two study groups were similar with regard to patient characteristics and parameters related to anesthesia and surgery. However, the incidence of successful anesthesia was significantly higher in obese patients. Furthermore, time to first report of postoperative pain and time to first self-void were significantly longer in obese patients. We also performed multivariate analysis to identify predictors of successful anesthesia. In addition to intrathecal bupivacaine dosage, higher BMI (≥ 30 kg/m^2^) was also associated with anesthesia success. Furthermore, BMI as a continuous variable was also associated with spinal anesthesia success.

There have been many studies on the influence of obesity on the spread of spinal anesthetics, but results remain conflicting [3–6,9,12–15]. The precise mechanism by which BMI affects the spread of a spinal block is unclear. However, cerebrospinal fluid (CSF) volume appears to be an important factor. Previous studies have speculated that CSF volume plays a major role [8,9] in determining spinal block level and duration. In a previous study [8], measured CSF volume correlated well with peak sensory block level in patients who received spinal anesthesia. Other patient characteristics, including age, weight, BMI, and height, were considered to be potential factors influencing inter-individual variation in CSF volume.

The positive relationship between obesity and distribution of sensory block has been evaluated in many studies [3–6,9,12,15]. A previous study demonstrated that CSF volume in obese patients is reduced by using magnetic resonance images [9]. Although the mechanism of CSF volume reduction in obese patients has not been proved, possible explanations suggest that reduced CSF volume is a result of increased intra-abdominal pressure or increased epidural fat [6,9,25]. If the inferior vena cava is occluded by increased abdominal pressure due to the weight of the abdominal contents in obese patients, blood flow increases through the lumbar vertebral plexus, and the extradural vein distends. Since a distended extradural vein is known to compress the CSF space, the volume of CSF is often reduced in obese patients, meaning that the spread of local anesthetics can be increased. If the same amount of bupivacaine is injected into a patient with a smaller CSF volume, the concentration of bupivacaine in the CSF would be higher, and a delayed regression of sensory block level needed for surgical coverage would be expected. In support of this idea, a previous study using myelography [12] demonstrated that abdominal compression increased the spread of radiopaque material in the CSF space. Thus, a greater cephalad spread of the sensory block level was achieved by increased intra-abdominal pressure. It is also theorized that the reduced CSF volume seen in obese patients may be due to increased extradural fact deposits, which could reduce CSF volume by compressing the dural sac [6]. However, a previous study with endoscopic observation of the epidural space reported that the amount of epidural fat did not appear to be correlated with BMI [26].

On the contrary, other studies have demonstrated that obesity does not influence spinal anesthesia. Recent studies have compared dose-response relationships in obese and non-obese patients [13,14], reporting that the ED_50_ for successful anesthesia in obese patients was not different from the ED_50_ in non-obese patients. However, these studies were performed under a different clinical setting and different definitions of successful anesthesia compared to those used in the present study. Successful anesthesia in these previous studies was defined as successful induction with no report of intraoperative surgical pain, and our definition emphasized the block level at the end of surgery. The different study methodologies appear to have led to discrepant results. In other words, spinal anesthesia can be significantly prolonged in obese patients, although the ED_50_ for obese patients may not be different from that of non-obese patients. The present study demonstrated the strong impact of obesity on the duration of spinal anesthesia and spinal anesthesia recovery.

Although vertebral column length has been reported to have a better correlation with level of sensory anesthesia than patient body weight or height [27], it has not yet been investigated whether vertebral column length is associated with CSF volume. However, vertebral column length was not an independent predictor of anesthesia success in this study.

In our study, we defined successful spinal anesthesia to compare anesthesia outcomes between obese and non-obese patients. A successful anesthesia induction was defined as that coupled with a sensory block ≥ the level of T12 with a sensory/motor block scale ≥ 2 at the end of the surgery. The sensory block level T12 was chosen because it can block both surgical (L3/4) and tourniquet pain (L1/2) until the end of surgery. Since the sensory block level regresses as time passes, the definition of anesthesia success in the present study is related to both spinal anesthesia duration and block height. However, our definition of successful anesthesia can also include spinal anesthesia with an excessively high block height. As such, we examined other variables related to anesthesia recovery including time to first-self void.

### Limitations

The present study did have some limitations. First, although this study was prospective, it was only observational. Although the demographic factors and bupivacaine dosages were not significantly different between the two groups, future study protocols should include a single, fixed dose of bupivacaine. Second, since no subject with a BMI >40 kg/m^2^ was enrolled in this study, the effect of morbid obesity [28] on spinal anesthesia was not evaluated. Furthermore, the effect of underweight with a BMI <18.5 kg/m^2^ on spinal anesthesia was not considered in this study. There were only two underweight patients in our study sample, which was not a sufficient number to be analyzed as a single study group. Third, the level of injection could have been higher than intended in obese patients [29], which might have produced higher sensory block levels than desired. Fourth, since we did not measure CSF volume or determine whether it was a potential predictor of anesthesia success, we cannot definitively conclude that obesity influences spinal anesthesia outcome due to smaller CSF volume. Fifth, since the patient group in our study was elderly with a large proportion of women, the external validity of our study is limited. Sixth, a single type of surgery was selected to compare the study endpoint under similar surgical condition.

### Conclusion

The duration of spinal block with hyperbaric bupivacaine appears to be prolonged in obese patients compared to non-obese patients. Multivariate analysis indicates that obesity, as well as bupivacaine dosage, are major predictors of spinal anesthesia outcome in patients undergoing TKRA. Given the high rates of obesity worldwide, it is important to determine the impact of obesity on anesthesia outcome. A future study using a single, fixed bupivacaine dosage administered to patients with morbid obesity is required to confirm the effect of obesity on spinal anesthesia. A study of this nature will also answer the question of whether or not bupivacaine dosages should be reduced in obese patients.

## Supporting Information

S1 File A dataset for the present study. (XLS)

S1 Table American Society of anesthesiologists (ASA) physical status classification. (DOC)

S2 Table Characteristics of spinal anesthesia according to four BMI categories. (DOC)

S3 Table Characteristics of spinal anesthesia according to the bupivacaine dosage. (DOC)
