# Supplementary material for: Obesity Is Independently Associated with Spinal Anesthesia Outcomes: A Prospective Observational Study
Source: PLoS One. 2015 Apr 21;10(4):e0124264. doi: 10.1371/journal.pone.0124264 (PMC4405588; doi:10.1371/journal.pone.0124264)
Supplement: S1 Table — (DOC) [file pone.0124264.s002.doc]

Table S1. American Society of anesthesiologists (ASA) physical status classification.

| Class | Physical status |
| --- | --- |
| Class 1 | Normal healthy patient. No organic, physiologic, biochemical, or psychiatric disturbance |
| Class 2 | Mild-to-moderate systemic disease that is well controlled and causes no organ dysfunction or functional limitation. |
| Class 3 | Severe systemic disease of at least one organ system that does cause functional limitation |
| Class 4 | Severe systemic end-stage disease of at least one organ system that is life threatening with or without surgery |
| Class 5 | A patient who has little chance of survival but is submitted to surgery as a resuscitative effort |
| Class 6 | A brain-dead patient whose organs are being removed for donor purposes |
| Class E | Any patient in whom an emergency operation is required |

Modified from American Society of Anesthesiologists; New classification of physical status. Anesthesiology 1963; 24: 111.
