# Supplementary material for: Obesity Is Independently Associated with Spinal Anesthesia Outcomes: A Prospective Observational Study
Source: PLoS One. 2015 Apr 21;10(4):e0124264. doi: 10.1371/journal.pone.0124264 (PMC4405588; doi:10.1371/journal.pone.0124264)
Supplement: S2 Table — (DOC) [file pone.0124264.s003.doc]

Table S2. Characteristics of spinal anesthesia according to four BMI categories.

| Variables | BMI < 25 | 25 ≤ BMI < 30 | 30 ≤ BMI < 35 | BMI ≥ 35 | p-value |
| --- | --- | --- | --- | --- | --- |
| Case number (n, %) | 61 (29.2%) | 80 (38.3%) | 49 (23.4%) | 19 (9.1%) |  |
| Bupivacaine dosage (mg) | 9 (7-10) | 8 (7-10) | 8 (7-10) | 8 (7-10) | 0.920 |
| Success/failure of anesthesia (n, n) | 43/18 (29.5%) | 55/25 (31.3%) | 39/10 (20.4%) | 19/0 (0%) | 0.012 |
| Success/failure of anesthesia induction (n, n) | 60/1 (1.6%) | 77/3 (3.8%) | 44/5 (10.2) | 19/0 (0%) | 0.012 |
| Time to first report of postoperative pain | 180 (140-205) | 180 (137-195) | 185 (165 – 200) | 193 (185 – 200) | 0.036 |
| Time to first self-void | 305 (280 – 338) | 290 (270 – 320) | 320 (290- 350) | 305 (300 – 330) | 0.002 |

The values are presented as the median (interquartile range), or the number of patients (%) per group.

P-values are the results of Kruskal-Wallis test or Fisher’s exact test.

BMI = body-mass index.
