# Supplementary material for: Obesity Is Independently Associated with Spinal Anesthesia Outcomes: A Prospective Observational Study
Source: PLoS One. 2015 Apr 21;10(4):e0124264. doi: 10.1371/journal.pone.0124264 (PMC4405588; doi:10.1371/journal.pone.0124264)
Supplement: S3 Table — (DOC) [file pone.0124264.s004.doc]

Table S3. Characteristics of spinal anesthesia according to the bupivacaine dosage.

| Bupivacaine dosage | 6 | 7 | 8 | 9 | 10 | 11 |
| --- | --- | --- | --- | --- | --- | --- |
| Case, n | 40 | 36 | 36 | 33 | 36 | 28 |
| Successful anesthesia, n (%) | 17 (42.5%) | 23 (63.9%) | 27 (75.0%) | 27 (81.8%) | 35 (97.2%) | 27 (96.4%) |
| Time to first report of postoperative pain, min | 133 (90-165) | 171 (133-185) | 185 (178-200) | 185 (170-200) | 203 (191-214) | 205 (199-222) |
| Time to first self-void, min | 279 (260-290) | 290 (272-306) | 301 (277-308) | 320 (284-345) | 335 (319-354) | 337 (316-361) |

The values are presented as the median (interquartile range), or the number of patients (%) per group.

.
